# Ten practical tips and tricks to improve the effectiveness of biological network alignment

**DOI:** 10.1371/journal.pcbi.1013386

**Published:** 2025-09-04

**Authors:** Giuseppe Agapito, Mario Cannataro, Pietro Cinaglia, Marianna Milano

**Affiliations:** 1 Department of Law, Economics and Social Sciences, Magna Graecia University of Catanzaro, Catanzaro, Italy; 2 Data Analytics Research Center, Magna Graecia University of Catanzaro, Catanzaro, Italy; 3 Laboratorio di Storia Giuridica ed Economica Research Center, Magna Graecia University of Catanzaro, Catanzaro, Italy; 4 Cultura Romana del Diritto e Sistemi Giuridici Contemporanei Research Center, Magna Graecia University of Catanzaro, Catanzaro, Italy; 5 Department of Medical and Surgical Sciences, Magna Graecia University of Catanzaro, Catanzaro, Italy; 6 Department of Health Sciences, Magna Graecia University of Catanzaro, Catanzaro, Italy; 7 Department of Experimental and Clinical Medicine, Magna Graecia University of Catanzaro, Catanzaro, Italy; SIB Swiss Institute of Bioinformatics, SWITZERLAND

## Abstract

Network alignment (NA) is a computational methodology employed to compare biological networks across different species or conditions. By identifying conserved structures, functions, and interactions, NA provides invaluable insights into shared biological processes, evolutionary relationships, and system-level behaviors. This manuscript presents a comprehensive overview of NA methodologies, including the importance of preprocessing network data, selecting suitable input formats, and understanding diverse network types such as attributed, temporal, and multilayer networks. Additionally, it explores key challenges such as seed nodes selection, algorithm configuration, and cross-species alignment, emphasizing the necessity of integrating functional annotations, sequence similarity, and network topology for biologically meaningful results. Various NA strategies, including Local and Global Network Alignment, are discussed alongside their respective advantages and limitations. Practical recommendations for effectively documenting and visualizing NA experiments are also provided, ensuring reproducibility and clarity in research. By leveraging diverse alignment tools and adopting best practices, researchers can unlock the potential of NA to advance our understanding of complex biological systems.

## Introduction

Network alignment [36] is a computational approach to compare biological networks across different species or conditions, such as protein-protein interaction networks, gene co-expression networks, or metabolic networks. NA aims to identify conserved substructures, functional modules, or interactions, providing insights into shared biological processes and evolutionary relationships [35].

Graph formalism is employed in biological networks to represent interactions or relationships between genes, proteins, or other molecular entities. In biological networks, genes, proteins, and any biological entity are represented using nodes, whereas interactions among biological entities are represented through edges. Several local and global algorithmic methods are available for network alignment, focusing on identifying sub network overlaps or conserved nodes while accounting for network structure and edge relationships [34]. For example, algorithms may optimize node and edge similarity, functional annotations, or topological features to generate alignments that maximize biological relevance—tools such as estimating the significance of conserved substructures [33].

Many NA tools are available as web-based platforms or standalone software. Some integrate multiple data sources, while others focus on specific network types, but all aim to compare input networks and identify significant conserved regions or interactions.

While NA can be performed relatively quickly using modern tools, avoiding common pitfalls, such as overinterpreting results or failing to account for network biases, is essential. Properly executed NA provides valuable insights into the functional and evolutionary relationships between biological systems, making it a powerful tool in systems biology. Formally, given two input networks *G*_1_ = (*V*_1_, *E*_1_) and $G_{2}=(V_{2},E_{2})$, the goal of NA is to find a mapping $f:V_{1}\to V_{2}$ ∪ {⊥}, where ⊥ represents unmatched nodes. The function *f* is optimized to maximize a similarity score based on topological properties, biological annotations, or sequence similarity. Intermediate steps of the NA process may include seed nodes selection, computation of similarity matrices, and iterative or heuristic optimization. The final output is a set of aligned node pairs or a similarity matrix highlighting conserved regions or functions across networks.

## Tip 1: Network node type and name nomenclature consistency

Ensuring consistency across node types beyond just gene names is critical for reliable network integration, comparison, analysis and alignment. Gene and protein nomenclature are interconnected, as names or identifiers used for a protein can often apply to its encoding gene and vice versa. Thus gene/protein name synonyms represent a significant challenge in bioinformatics and genetics research. Synonyms refer to different names or identifiers that are used to describe the same gene and/or protein across various databases, publications, and studies [10]. This situation arises due to a lack of standardized nomenclature in the early stages of genetic research and the continuing discovery and renaming based on gene function, structure, or disease association. Moreover, variations in genomic coordinates across assemblies or species, as well as the use of different protein identifiers (e.g., UniProtKB vs. NCBI RefSeq), can significantly affect data harmonization.

Here are the critical problems associated with node name synonyms that complicate matching the same node:

- **Data Integration** complicates the attempt to integrate data from multiple sources necessary for the researcher. Synonyms can make it challenging to establish if the data refer to the same gene, protein, genomic region, even for automated systems, leading to redundancy, inconsistencies, and errors in the integrated datasets.
- **Genomic databases** may use different primary names for genes or proteins. Users querying these databases must be aware of all potential synonyms to ensure they retrieve all relevant data.
- **Bioinformatics tools** and software designed to analyze genetic data may not recognize synonyms, leading to incomplete or inaccurate analyses, affecting the accuracy and completeness of analysis.

Various strategies have been employed to address genetic research challenges associated with gene name synonyms. Organizations like the HUGO Gene Nomenclature Committee (HGNC) [11] have developed standardized nomenclature systems for human genes. Bioinformatics tools and databases also have synonym mapping features to help users navigate the complexity of gene nomenclature. Despite these efforts, gene name synonyms still pose a significant hurdle in genetic research. Performing input data harmonization before NA is crucial for researchers. This ensures that the data is properly adjusted and ready for alignment.

**Practical Recommendations**: To ensure consistent and accurate network alignment, we recommend the following preprocessing steps:

- Incorporating ad-hoc robust identifier mapping and normalization strategies—such as leveraging cross-references provided by resources like UniProt, HGNC, or Ensemble.
- Normalize gene names across datasets using tools such as UniProt ID mapping, NCBI Gene, or MyGene.info API.
- Where possible, adopt HGNC-approved gene symbols for human datasets and equivalent authoritative sources for other species (e.g., MGI for mouse).
- Use programmatic mapping tools such as BioMart (Ensembl), R packages like biomaRt, or Python APIs to unify identifiers before network construction.

Reconciling these discrepancies can help to improve the accuracy of multi-source or cross-species network analyses.

**Example Workflow**:

1. Extract all gene names or IDs from your input networks.
2. Query a gene ID conversion service (e.g., UniProt, BioMart) to retrieve standardized names and known synonyms.
3. Replace all node identifiers with the standard gene symbol or ID.
4. Remove any duplicate nodes or edges introduced by merging synonyms.

**Why this matters**: Modern alignment tools may rely on exact node name matching. Failure to harmonize gene names leads to:

- Missed alignments of biologically identical nodes
- Artificial inflation of network size and sparsity
- Reduced interpretability of conserved substructures

In summary, harmonizing gene identifiers is a simple yet essential step in ensuring accurate and reproducible biological NA. Incorporating these best practices minimizes semantic ambiguity and enhances the quality of downstream results.

## Tip 2: Network structure types and models

NA is fundamentally connected to the type of representation used to model networks, as the chosen representation determines how structural and functional features of the networks are captured, processed, and compared. The way a network is represented—whether through adjacency matrices, edge lists, or compact sparse matrix representation format—directly impacts the effectiveness and efficiency of NA.

Different representations encode network features in distinct ways. For example, adjacency matrices directly represent connectivity between nodes. Still, they can be computationally expensive for large and sparse networks; instead, edge lists represent the set of connections or relationships between nodes, making them more suitable to represent large networks.

The choice of representation also significantly impacts computational efficiency. The adjacency matrix represents the network and provides insight into the presence or absence of an edge between each pair. While adjacency matrices are more comprehensive and facilitate fast lookups of connections, they can become memory-intensive, especially for large, sparse networks, where many entries in the matrix remain unused. Adjacency matrices can be computationally expensive to manipulate large-scale networks, especially if the network representation results in a sparse matrix. In this manner, using a specific format such as YALE even known as compressed sparse row (CSR) [37], focused on representing only the non-zero values, reduces memory consumption, making alignment tasks more easily feasible. In addition, an edge list explicitly provides information about the neighborhood of each node, in a compact and efficient manner even to represent networks as sparse matrices, but in the same time is less effective for computing.

Finally, the representation type influences the NA process’s accuracy and computational feasibility.

NA is intricately connected to network representation; thus, selecting the most proper representation format is crucial to ensuring the alignment algorithm achieves its goals efficiently and effectively. Table 1 outlines the strengths and limitations of each representation format for modeling different types of biological networks.

**Table 1:** Comparison of network representation formats.

Format	Advantages	Disadvantages	Use Cases
Adjacency Matrix	Easy to query connections; Comprehensive representation	Memory-intensive for large sparse networks	Small, dense networks
Edge List	Compact; Suitable for large sparse networks	Less efficient for computational queries	Large-scale networks
Compact Sparse Matrix	Reduces memory consumption; Optimized for sparse data	Requires specialized handling	Large-scale, sparse networks

The Table 2 summarizes the most suitable network representation formats for a particular type of biological network, along with the rationale for each choice.

**Table 2:** Recommended network representation formats for different types of biological networks. Each biological network type exhibits unique topological characteristics that make certain representation formats more efficient for storage, analysis, or alignment.

Biological network type	Preferred representation	Justification
Protein–Protein Interaction (PPI)	Adjacency list	Typically large and sparse; adjacency lists are memory-efficient and support scalable traversal.
Gene Regulatory Network (GRN)	Adjacency matrix	Dense interactions benefit from matrix-based operations and compact representation of pairwise relationships.
Metabolic Network	Edge list	Often directed and weighted; edge lists offer flexible parsing and preserve path directionality.
Co-expression Network	Adjacency list	Usually sparse with modular structure; adjacency lists support efficient neighborhood exploration.
Signaling Network	Adjacency matrix	Captures complex regulatory relationships; matrices support algorithmic operations and fast lookups.

**Fig 1:**
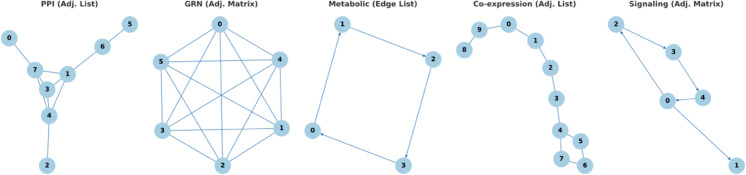
Network topologies and suitable representation formats. Illustrations of common biological network types and their recommended data representations. The choice of format depends on network structure and influences the efficiency of alignment and analysis.

Fig 1 illustrates the topological features of each network type and highlights the preferred representation format.

## Tip 3: Input data types and formats

The structure and representation of network data play a pivotal role in shaping the Network Alignment (NA) process and its outcomes. Since NA aims to identify meaningful similarities between the nodes and edges of two or more networks, understanding the structural format and characteristics of these networks is essential.

To enhance clarity, we distinguish here between two related but distinct concepts: (i) the biological data type, which refers to the nature of entities and interactions (e.g., protein-protein interactions, gene regulation, metabolic pathways), and (ii) the network representation format, which refers to the computational encoding of such data (e.g., graph, adjacency matrix, knowledge graph).

The network representation format directly influences which features can be extracted and leveraged during NA. Biological networks encode both functional annotations and topological patterns, which in turn guide similarity assessments and alignment strategies. For example, the use of attributed graphs or multilayer networks enables the inclusion of richer biological context, improving the interpretability and precision of alignment results.

Below, we describe common network representation formats relevant for biological data:

- **Graph:** The fundamental data structure where nodes represent entities (e.g., genes, proteins) and edges represent interactions (e.g., regulatory links, binding events). Graphs can be undirected or directed, and weighted or unweighted. They are widely used across biological domains.
- **Attributed Networks:** These extend basic graphs by associating additional metadata to nodes and edges. Node attributes may include functional categories or gene ontology terms; edge attributes may reflect interaction strength or evidence level. This format enhances alignment by incorporating semantic detail.
- **Knowledge Graphs:** A specialized form of attributed network in which edges are labeled relationships, often drawn from ontologies. These graphs are especially useful in tasks requiring semantic integration, such as aligning biological data with knowledge bases.
- **Multilayer Networks:** Consist of multiple interrelated layers, each encoding a different interaction type (e.g., protein interactions in one layer, metabolic reactions in another). This allows the modeling of heterogeneous and interdependent biological systems.
- **Temporal Networks:** A dynamic variant of multilayer networks in which layers represent time steps. Nodes and edges can appear or disappear over time, capturing temporal evolution of biological processes such as disease progression.
- **Hypergraphs:** Extend traditional graphs by allowing hyperedges to connect more than two nodes, enabling the representation of higher-order interactions such as protein complexes or gene modules.

**Fig 2:**
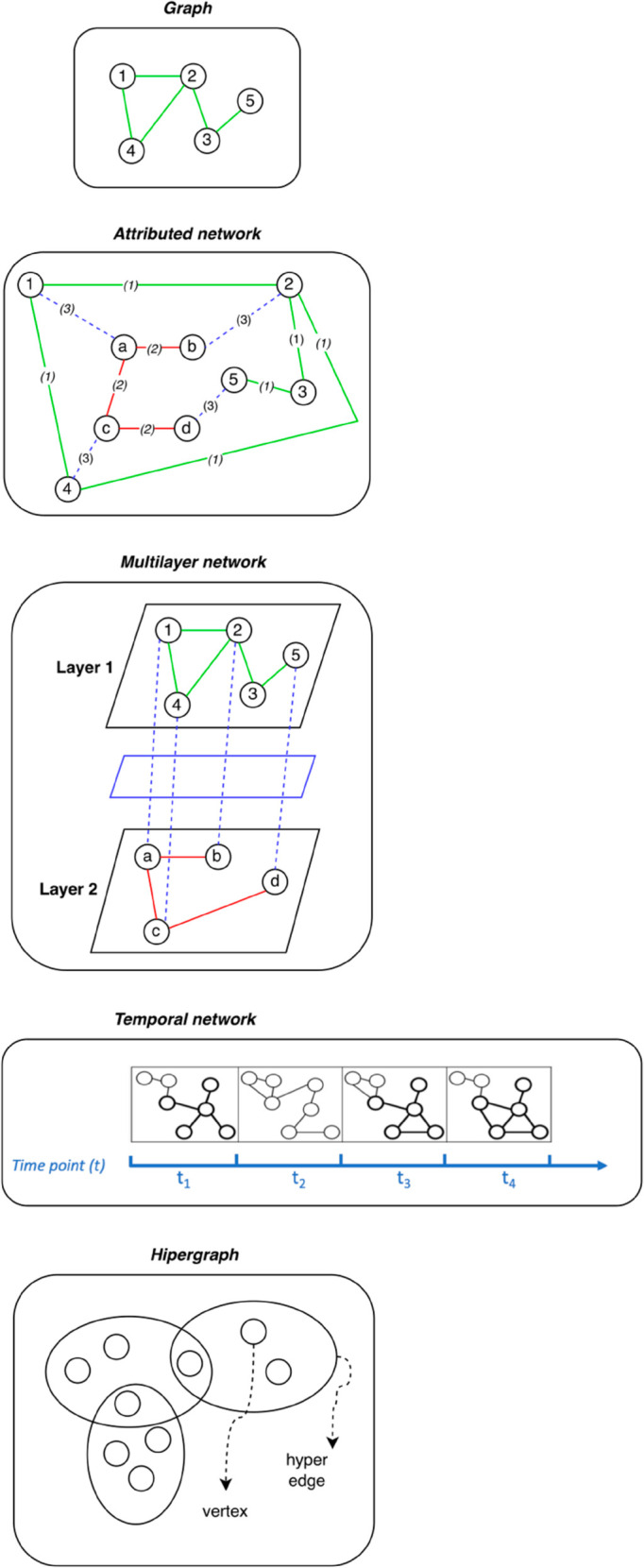
This figure shows a non-exhaustive representation of the main models reported in our discussion, for illustrative purpose only. Note that it omitted Knowledge graph by including its own representation within attributed networks, being the former a specialized form of the latter.

Fig 2 shows a non-exhaustive representation of the main models reported in our discussion, for illustrative purpose only. It unites in a single representation Attributed networks and Knowledge graphs, being the latter a specialized form of the former.

The chosen representation format also determines the type of similarity measures applicable to the NA task. Multilayer and temporal networks, which integrate heterogeneous or time-resolved data, require more complex, context-aware similarity metrics. In contrast, homogeneous networks, such as co-expression graphs, may suffice with standard similarity functions.

Ultimately, a deep understanding of the input data’s structure and semantics is crucial for selecting or designing NA algorithms that are both computationally efficient and biologically meaningful.

Deep knowledge of the input data is essential for selecting or designing NA algorithms that are both effective and computationally efficient, ensuring that the results are meaningful and interpretable within the context of the specific study. A comparative summary of these formats, along with their features and applications, is provided in Table 3.

**Table 3:** Summary of input data types and their characteristics.

Data Type	Description	Applications and Advantages
Graph	Nodes represent entities (e.g., proteins, genes); edges represent interactions (e.g., protein-protein, gene-mRNA). Graphs can be undirected or directed, weighted or unweighted, or a combination of these characteristics.	Common in biological networks; weighted graphs highlight interaction strengths.
Attributed Networks	Extend graphs by including additional information like node attributes (e.g., functional annotations, labels) and edge attributes (e.g., interaction types, confidence scores).	Improve accuracy by adding contextual data; useful for biological networks.
Multilayer Networks	Consist of interconnected layers, each representing a distinct type of relationship (e.g., protein interactions, metabolic pathways).	Capture complex real-world systems, ideal for systems biology.
Temporal Networks	Model time series data with dynamic nodes and edges that evolve over time.	Align entities based on evolving interaction patterns in biological data.
Hypergraphs	Represent higher-order relationships where edges (hyperedges) connect more than two nodes.	Useful for domains like group collaborations or multi-component protein complexes.
Knowledge Graphs	Specialized attributed networks with labeled edges enriched with semantic information.	Used in ontology-based tasks and natural language processing.

## Tip 4: Network data preprocessing

Network data preprocessing is an essential step in yielding high-quality NA results. Data preprocessing allows the removal or reduction of inaccuracy, inconsistency, and noise in the data, which can create biases in NA results.

Preprocessing provides many methodologies to deal with data quality issues, including both missing data and outliers. These are often incorrectly treated as equivalent, but in reality refer to distinct problems. **Missing data** occurs when certain nodes, edges, or attributes are entirely absent from the dataset due to experimental limitations or incomplete annotations. This can be addressed through imputation methods or by integrating external data sources. **Outlier removal**, on the other hand, focuses on eliminating extreme or anomalous values (e.g., nodes with disproportionately high degree or confidence scores) that can distort the topology or introduce bias in the alignment process. These two strategies are complementary and should be applied depending on the specific characteristics of the input networks.

Networks often present differences in **size** and **scale**, which can distort NA outcomes if not addressed properly. In this context, **size** refers to the number of nodes and edges in a network—essentially its cardinality—while **scale** refers to the statistical distribution and density of connections (e.g., node degree distribution, presence of hubs, or modularity patterns). Two networks may be similar in size but differ dramatically in scale, which would affect alignment strategies. Using normalization as a preprocessing step makes it possible to increase the compatibility of the two networks and, subsequently, the quality of NA results.

For example, degree normalization adjusts for the differences in node connectivity, making comparing networks with varying densities easier. Attribute scaling can be used as preprocessing when it is necessary to align networks enriched with node or edge attributes, ensuring that these features contribute appropriately to the alignment. In addition, edge weighting is particularly relevant in biological networks, where interactions with high confidence scores should be emphasized over less reliable ones. Incorporating such domain-specific insights during preprocessing improves the relevance and interpretability of the alignment results.

Network representation impacts the NA performance due to the high dimensional spaces used to represent biological networks. Graph embeddings and/or vector representations can be used as preprocessing steps to move networks from high to low-dimensional spaces without losing structural and attribute information. Graph embeddings and vector representation reduce the computational complexity of traditional NA algorithms, enabling faster and more accurate alignments. Table 4 provides a schematic comparison among the preprocessing methods for NA.

**Table 4:** Comparison of preprocessing methods for network alignment.

Method	Purpose	Advantages	Limitations
Normalization	Adjusts for differences in size, scale, and connectivity	Improves compatibility; Enhances quality of results	May oversimplify complex networks
Attribute Scaling	Aligns enriched networks with attributes	Ensures attributes contribute effectively	Requires attribute-specific preprocessing
Edge Weighting	Highlights high-confidence interactions	Improves biological relevance	Dependent on reliable confidence scores
Graph Embeddings	Reduces dimensionality while retaining structure	Reduces computational complexity	Can lose fine-grained details

In conclusion, the preprocessing methods should be chosen based on the kind of networks used to analyze and the specific alignment task. For example, aligning biological pathways might prioritize functional annotation and interaction confidence scores, whereas protein interaction networks may require preprocessing steps focused on community detection and behavioral patterns.

## Tip 5: Cross-species networks

To be effective in investigating networks from different organisms, network alignment (NA) must address evolutionary divergence and biological heterogeneity across species. A fundamental strategy in cross-species NA is the use of orthology information, which helps identify genes or proteins that have evolved from a common ancestral gene and are likely to perform similar functions. Orthologs can be identified using tools and databases such as OrthoDB [12] or InParanoid [13], which support the annotation of evolutionarily conserved entities across species. Incorporating orthology information guides the alignment process by establishing biologically grounded node correspondences between networks, thus improving the accuracy and reliability of alignment results. Functional annotations contribute to highlighting shared biological roles between nodes in different networks, allowing the alignment process to focus on conserved functions even when direct topological similarity is weak or absent. These annotations, such as Gene Ontology (GO) terms, can be compared using semantic similarity metrics like Resnik, Lin, or Jiang-Conrath scores, which quantify the functional closeness between two proteins or genes in terms of shared biological meaning [3, 4]. To further enhance biological validity, functional annotations are often combined with sequence similarity scores obtained through tools like BLAST [5], which identify homologous proteins based on conserved amino acid sequences. However, relying solely on sequence alignment may miss functionally relevant but structurally divergent regions. Therefore, many NA algorithms adopt hybrid approaches that integrate functional, sequence, and topological similarities—such as node centrality, clustering coefficients, or graphlet-based measures [6, 7]. These diverse similarity signals can be combined using composite scoring functions or multi-objective optimization. For example, IsoRank [8] uses spectral methods to combine topology and sequence similarity, while HubAlign [9] emphasizes high-centrality nodes to enhance biological relevance. Multilayer alignment tools like those presented in [38] further improve robustness by integrating heterogeneous network types (e.g., protein-protein interactions, co-expression, metabolic pathways) into a single alignment model. Why does evolutionary information improve scalability? Incorporating orthologous relationships or sequence similarity as a preprocessing step significantly reduces the search space for alignment by narrowing down the set of candidate node pairs to those that are biologically plausible. Instead of comparing all nodes exhaustively (quadratic complexity), alignment algorithms can prioritize orthologous pairs, leading to more scalable and efficient computations. For instance, an initial seed set defined by orthologs helps constrain optimization and focus alignment on the most promising regions of the network. In summary, functional annotations serve as a semantic bridge between species, sequence similarity ensures evolutionary plausibility, and topological features support structural coherence. Their integrated use enhances both the biological accuracy and the computational feasibility of cross-species network alignment. These strategies are particularly important when aligning networks from evolutionarily distant species, where topological noise and functional divergence are most pronounced.

## Tip 6: Network alignment algorithms

The comparison of conserved substructures across species provides critical insights into complex biochemical processes. However, aligning biological networks remains a computationally demanding task due to their topological complexity and the intractability of many underlying problems. Existing network alignment (NA) algorithms are typically classified into two categories: Local Network Alignment (LNA) and Global Network Alignment (GNA). **Local Network Alignment (LNA)** aims to identify small, highly similar subnetworks—such as conserved motifs or functional modules—shared across input networks. LNAs detect multiple, potentially disjoint regions of isomorphism, with each region comprising nodes of maximal similarity. Unlike GNAs, which align networks in their entirety, LNAs focus on localized, functionally relevant similarities. Two primary strategies are employed in LNA: (1) *Mine and Merge*, where subnetworks are extracted independently from each input and subsequently aligned; and (2) *Merge and Mine*, which integrates the input into a single alignment graph for analysis. The former is less computationally intensive but more sensitive to noise and redundancy, while the latter offers greater robustness at a higher computational cost. **Global Network Alignment (GNA)**, by contrast, constructs a one-to-one mapping between the nodes of two networks, aiming for a comprehensive alignment that covers their full topology. GNAs are particularly useful for tasks such as cross-species knowledge transfer and comparative interactomics, though they may overlook small but functionally important modules. Table 5 summarizes the key differences between LNA and GNA approaches.

**Table 5:** Comparison of local and global network alignment approaches.

Alignment Type	Description	Advantages and Limitations
Local Network Alignment (LNA)	Identifies small, conserved subnetworks such as motifs or functional blocks. Includes *Mine and Merge* and *Merge and Mine* strategies.	**Advantages:** Effective for detecting conserved patterns.
		**Limitations:** Sensitive to noise and input redundancy; merge-based methods improve accuracy but require more computation.
Global Network Alignment (GNA)	Constructs a full-scale mapping between all nodes in the networks.	**Advantages:** Enables comprehensive comparisons and knowledge transfer.
		**Limitations:** May miss localized features; computationally expensive.

Numerous tools support LNA, GNA, or both. Prominent GNA tools include IsoRank [8], MAGNA++ [15], and HubAlign [9], which integrate topological and biological data for full-network alignment. LNA-focused tools, such as AlignMCL [28] and SPINAL [27], specialize in identifying functionally conserved regions. Hybrid approaches, like DeepAlign [22], employ machine learning to balance local and global alignment objectives. Tool selection should be guided by the nature of the input networks and the goals of the analysis, as methods differ in scalability, biological fidelity, and computational cost. Among recent GNA algorithms, SANA (Simulated Annealing Network Aligner) [16] has demonstrated superior performance across multiple benchmarks. Unlike methods constrained to predefined scoring schemes, SANA allows users to optimize arbitrary objective functions, including both topological metrics (e.g., edge correctness) and functional similarity (e.g., GO term alignment). This flexibility enhances its applicability to diverse experimental contexts. In a comparative study [17], SANA consistently outperformed competing methods, establishing itself as a robust and adaptable GNA tool. In summary, LNA techniques are particularly valuable for detecting conserved functional subnetworks but face limitations related to initialization, noise resilience, and computational efficiency. These challenges underscore the need for continued development of alignment algorithms that are both biologically accurate and computationally scalable.

## Tip 7: Seed nodes selection

A common challenge in Network Alignment (NA) methods lies in the selection of seed nodes, representing the starting points for the alignment process.

Without predefined seed nodes, NA must explore a combinatorially large space of possible node mappings, making the task computationally expensive and resulting in scalability challenges. To illustrate, this can be likened to computing the Cartesian product of two node sets. Moreover, when seed nodes are used, their identification typically relies on external data sources, such as orthology databases, or on computational tools that estimate sequence or semantic similarity to infer candidate orthologous pairs.

The alignment results can be significantly impacted if seed node information is incomplete and/or inaccurate. Although seed nodes are commonly used to guide NA based on known correspondences such as orthologs or functionally related entities, they are not strictly required. In fact, relying too heavily on seed nodes can introduce bias—especially when the external annotations used to identify them are incomplete, inconsistent, or outdated. To mitigate this, several unsupervised NA methods avoid using predefined seed nodes altogether. These approaches rely instead on intrinsic properties of the networks—such as topological similarity or node embeddings—to infer meaningful correspondences in a data-driven manner. Avoiding seed nodes can thus reduce reliance on external databases and improve alignment robustness, particularly in poorly annotated or cross-species datasets.

The selection of seed nodes for biological NA can be broadly outlined in a series of general steps.

- Identify Candidate Node Pairs:
- sequence similarity, use BLAST to compute sequence alignment scores;
- functional annotations, match nodes based on shared functional attributes, such as Gene Ontology terms or pathway membership;
- network topology, use degree, and clustering coefficient to analyze network features to identify nodes with comparable roles in their respective networks.
- Rank Candidate Pairs Score: rank node pairs based on combined similarity metrics and weighted evidence.
- Select Initial Seed Nodes: Choose high-confidence pairs that are biologically meaningful and well-distributed across the networks, e.g., choose pairs with the highest scores as initial seeds.

These general steps provide a flexible way for selecting seed nodes in biological NA. Table 6 describes a comparison among the node selection methods.

**Table 6:** Comparison of seed node selection methods.

Criterion	Description	Advantages	Limitations
Sequence Similarity	Matches based on evolutionary conservation	Biologically meaningful	Requires reliable sequence data
Functional Annotations	Matches based on shared roles or pathways	Captures biological relevance	Dependent on annotation quality
Network Topology	Uses degree and clustering for comparable roles	Independent of external data	Sensitive to noise
Hybrid Methods	Combines multiple criteria	Improves robustness	Computationally intensive

In this context, the node embeddings and the topological analysis between pairs of networks can be applied for overcoming existing seed nodes, by inferring the pairwise similarities between nodes of two different networks. For instance, the representative learning of node features (i.e., node embeddings) can be used constructing the resulting similarity matrix of interest [23], on which a network alignment algorithm can be applied, directly.

## Tip 8: Network alignment algorithms configuration

Network alignment is a critical task in computational biology and network science, aimed at mapping nodes between networks to uncover structural and functional correspondences. This challenge is particularly significant in the context of biological data modeling, where the alignment of molecular interaction networks, such as protein-protein interaction (PPI) or metabolic networks, can reveal conserved pathways, functional modules, and evolutionary relationships. The configuration of alignment algorithms for such applications must carefully balance computational feasibility and biological relevance, given the inherent complexity of the problem. As an NP-hard problem, optimal solutions for NA are computationally prohibitive, especially for large-scale biological networks. To address this, heuristic approaches are widely adopted to design scalable algorithms that approximate solutions while retaining biologically meaningful insights. These heuristics often integrate biological data-specific features, such as sequence similarity or functional annotations, with structural properties of the networks. Methods such as local optimization, probabilistic models, or machine learning-based strategies provide practical means to align networks, enabling insights that would be computationally unattainable with classical methods alone. Through these tailored configurations, heuristic-driven alignment algorithms play a pivotal role in advancing our understanding of complex biological systems.

Several heuristic approaches have been developed to overcome the computational challenges posed by NA, especially in the context of large-scale biological networks. These methods offer approximate solutions that balance biological interpretability with computational efficiency. Heuristics can be categorized based on their underlying principles, such as local optimization, probabilistic models, evolutionary strategies, or machine learning-based techniques. Each approach presents different trade-offs between runtime, accuracy, and scalability. To provide a clearer overview of these strategies, Table 7 summarizes the main types of heuristics used in NA, highlighting their key advantages and limitations. This structured comparison can help researchers select the most appropriate strategy based on the characteristics of their data and the goals of their alignment tasks.

**Table 7:** Heuristic approaches for configuring network alignment algorithms.

Approach Type	Description	Advantages	Limitations
Local Optimization	Iteratively refines alignment to improve local matches.	Scalable; relatively simple to implement.	Prone to local optima; quality depends on initialization.
Probabilistic Models	Use random walks or stochastic processes to explore node similarity.	Can capture complex relationships; explores larger solution space.	Results may be variable; high computational cost.
Machine Learning	Learns alignment scores from node and edge features using supervised or unsupervised learning.	High accuracy; adaptable to heterogeneous data.	Requires labeled data or feature engineering; may be harder to interpret.
Evolutionary Algorithms	Mimic biological evolution using populations, crossover, and mutation to find optimal alignments.	Robust global search; avoids local minima.	May require many iterations; parameter tuning can be complex.

Several software tools have successfully implemented these heuristics to address scalability. For instance, MAGNA++ [15] uses a genetic algorithm to optimize node and edge conservation in global alignments. HubAlign [9] applies a centrality-based heuristic to reduce search space and speed up computations while preserving biologically relevant nodes. GHOST [14] leverages multiscale spectral signatures for scalable alignment. Machine learning-based approaches such as DeepAlign [22] predict node correspondence using learned representations, effectively managing both accuracy and scalability. These tools demonstrate the practical effectiveness of heuristic techniques and provide concrete references for researchers designing scalable NA methods. Table 8

**Table 8:** Representative tools using heuristic strategies for network alignment.

Tool	Heuristic Type	Advantages	Limitations
MAGNA++ [15]	Genetic Algorithms	Balances node and edge conservation; good for global alignment.	High computational cost for large networks.
HubAlign [9]	Centrality-based Heuristic	Fast and scalable; prioritizes important nodes.	May neglect biologically relevant peripheral nodes.
GHOST [14]	Spectral Signatures	Captures structural patterns efficiently; scalable.	Less flexible for heterogeneous data.
DeepAlign [22]	Machine Learning	Learns complex features; adapts to diverse data types.	Requires training data and computational resources.

Another key consideration in configuring NA algorithms is the selection of an appropriate objective function. The objective function determines what the alignment process will prioritize—whether it be topological features (e.g., edge conservation, graphlet similarity), biological consistency (e.g., GO term similarity), or a hybrid combination of both. The effectiveness of an alignment algorithm is strongly influenced by this choice, as different objective functions may lead to different alignment results even when applied to the same networks.

Rather than relying on a single, fixed objective function, we strongly recommend evaluating multiple options empirically. For instance, structural objectives are ideal when the goal is to compare network architecture, while biological objectives may be better suited for discovering functionally conserved modules. Some alignment frameworks, such as SANA [16], provide the flexibility to test various objective functions by allowing the user to define and optimize a custom score. This enables researchers to tailor the alignment process to their specific biological or computational questions.

In practice, testing and comparing several objective functions on the same dataset provides insight into how alignment priorities impact the final results. We encourage users to report both alignment performance and biological interpretability under different scoring criteria, thereby improving transparency and reproducibility of NA studies.

## Tip 9: GNA and LNA need dedicated tools, don’t confuse them

Several NA software tools have been developed to promote the investigation of biological networks, each employing distinct methodologies and features tailored to reply to specific research questions. These tools use advanced algorithms to identify conserved substructures, interactions, or functional modules across networks, revealing insights into biological processes and evolutionary relationships.

Alignment approaches can be categorized into two main classes: Global NA (GNA) and Local NA (LNA).

Regarding to GNA, we report a set of well-known tools for information purposes.

- IsoRank [21], which uses k-partite matching to extract the final global alignment across all the species.
- IsoRankN [32] employs spectral methods to align entire networks by maximizing the consistency of node matches based on topological similarity and sequence homology.
- MAGNA++ [31] employs genetic algorithms to optimize alignment quality, offering flexibility for various network types and metrics.
- NETAL [30] is based on an iterative approach for computing alignments that prioritizes overall topological similarity.
- HubAlign [29] employs a hub-based strategy, prioritizing nodes with high centrality to improve alignment performance for scale-free networks.
- GRAAL [25] that perform automatically several similarity measures to achieve the more comprehensive alignment results.

In recent years, other solutions have been designed for GNA, in order to extend the type of network models supported in computation. For instance, networks that evolve over time (i.e., dynamic and temporal networks) and networks modeled over multiple layer of interest (i.e., multilayer networks) are supported by DANTE [38] and DANTEml [24], respectively.

These are able to infer the similarity matrix based on topological features, overcoming the issue discussed in Tip 7. In both cases, the alignment strategy is GNA, which produces a one-to-one node mapping.

Belong to the LNA, tools such as AlignMCL [28] focus on identifying conserved sub network clusters by combining network topology with functional data. SPINAL [27] applies heuristics to identify local alignments without compromising accuracy.

Recently, NA approaches have been evolved by integrating deep learning. Tools like DeepAlign [22] use deep learning models to predict the alignments, in order to enhance results’ accuracy and precision. SUMONA [26] is based on OptNetAlign algorithm to create a population of alignments obtained using Uniform Partially Matched Crossover (UPMX), mutation and local search operations based on an efficient swap method.

The identification of the appropriate tool requires careful consideration of the research objectives, the type of networks being analyzed, and the desired level of resolution. Therefore, we reported an overview of the main features of the mentioned software tools in Table 9.

**Table 9:** Overview of the main features of the mentioned software tools. “*” represents a feature that has not been possible to evaluate.

Tool	Language	Input	Output	Key Features
Global Alignment Tools				
IsoRank [21]	C++	Graphs (Adjacency Matrices)	Node Correspondences	Uses spectral methods to align networks based on node similarity.
IsoRankN [32]	C++	Graphs	Multiple Network Alignments	Extension of IsoRank, supports multiple network alignment.
MAGNA++ [31]	C++	Graphs (Edge Lists)	Optimized Network Alignment	Uses genetic algorithms to optimize alignment scores.
NETAL [30]	C++	Graphs	Aligned Network	Uses topological similarity for alignment. Not available for download.
HubAlign [29]	C++	Graphs (Adjacency Matrices)	Node Correspondences	Prioritizes hubs (high-degree nodes) in alignment.
GRAAL [25]	C++	Graphs	Aligned Network	Uses graphlet-based similarity measures.
DANTE [38]	Java	Graphs	Optimal Alignment Mapping	Uses integer programming for optimal alignment.
DANTEml [24]	Java	Graphs	Machine Learning-Based Alignment	Machine learning extension of DANTE.
**Local Alignment Tools**				
AlignMCL [28]	Python	Graphs	Clustered Alignments	Uses Markov clustering to detect local alignments.
SPINAL [27]	C++	Graphs	Local Node Correspondences	Uses sequence and topological similarity for local alignment.
**Network Alignment Tools Integrating Deep Learning**				
DeepAlign [22]	C++	Graphs	Deep Learning-Based Alignment	Uses deep learning for similarity learning in network alignment.
SUMONA [26]	*	Graphs	Enhanced Alignment Mapping	Integrates deep learning to improve alignment accuracy.

## Tip 10: Describe all your NA tests and their details

Proper NA documentation enables reproducibility, transparency, and effective communication of discoveries. Fundamental aspects to document include the study’s objectives, input data, experimental setup, alignment criteria, results, and analysis. Comprehensive documentation guarantees that experiments can be replicated, limitations are identified, and the relevance of insights is improved.

- Study Context: Clearly define the purpose of the alignment and its biological or computational goals, such as identifying conserved pathways or evolutionary relationships.
- Input Data and Preprocessing: Describe the type and source of networks (e.g., protein-protein interaction networks) and detail preprocessing steps like cleaning, normalization, or handling missing data.
- Experimental Setup: Specify the algorithms used, parameter settings, and computational resources. Include information about software versions, libraries, and any parallelization strategies.
- Seed Node Selection and Criteria: Document the methods for selecting seed nodes (e.g., based on sequence similarity or network topology) and the metrics used for alignment.
- Results and Analysis: Present alignment outcomes with metrics like alignment scores and coverage. Use visualizations to improve interpretation of results, highlighting both biological relevance and performance.
- Reproducibility: Share code and datasets through public repositories and acknowledge limitations or biases.

**Tip:** To improve workflow clarity and reproducibility, we recommend using structured computational notebooks (e.g., Jupyter, RMarkdown) or pipeline tools (e.g., Snakemake, Nextflow). These frameworks allow clear tracing of tests and alignment steps.

Follows a practical example of NA documentation.

Suppose that we have to align Homo sapiens and mouse protein-protein interaction (PPI) networks to identify conserved functional modules. The documentation should describe the following relevant aspects.

- Context: The goal is to discover conserved protein complexes between humans and mice, focusing on shared biological processes, such as metabolic pathways.
- Input Data: The human PPI network is sourced from BioGRID, while the mouse network is from STRING. Both networks are cleaned by removing nodes with low connectivity and normalizing them to the node degree.
- Experimental Setup: A heuristic-based Global Network Alignment (GNA) algorithm, such as IsoRank, is used. The implementation is in Python using NetworkX and NumPy, executed on a server with 32 CPUs and 128GB RAM.
- Seed Node Selection: Orthologs are identified using OrthoDB. Sequence similarity scores from BLAST are used to prioritize seed pairs, supplemented by functional annotations from Gene Ontology (GO). Orthologs are enriched using pathway enrichment tools such as BiP [20], PathDIP [19], and GSEA [18].
- Results: The alignment yields an overall coverage of 78%, with conserved subnetworks including key metabolic enzymes. Visualization in Cytoscape [2] or NAViGaTOR [1] reveals clusters corresponding to known protein complexes.
- Reproducibility: Scripts for preprocessing, alignment, and visualization are shared on GitHub, along with data and configuration files. Limitations, such as incomplete GO annotations, are acknowledged.

This example illustrates how systematic documentation clarifies the NA process and enhances the reproducibility and impact of NA research.

Table 10 highlights the key aspects to describe when performing NA.

**Table 10:** Key aspects of network alignment documentation.

Aspect	Description
Study Context	Clearly define the purpose, objectives, and hypotheses of the NA study. Specify the biological or computational questions being addressed, such as identifying conserved pathways or functional modules.
Input Data	Provide detailed information about the source, type, and structure of the networks (e.g., PPI networks, metabolic pathways). Include details on data cleaning, normalization, and handling of missing information.
Experimental Setup	Document the algorithms used (e.g., GNA, LNA), their configurations, parameter settings, and versions. Specify the computational resources (e.g., CPU, GPU, memory) and software environments.
Seed Node Selection	Describe the criteria for selecting seed nodes, such as sequence similarity, functional annotations, or topological properties. Specify tools or databases (e.g., BLAST, OrthoDB) used in the selection process.
Alignment Criteria	Define the metrics used to evaluate node and edge similarities (e.g., sequence identity, semantic similarity). Specify how multiple criteria are weighted or combined if applicable.
Results and Analysis	Present outcomes with key metrics like alignment scores, runtime, and coverage. Include visualizations, such as graphs or heatmaps, to interpret the results. Highlight biological relevance and computational efficiency.
Reproducibility	Share all scripts, code, and datasets in public repositories. Document software dependencies and versions. Acknowledge limitations and potential biases in the study.

## Conclusion

Network alignment is a pivotal technique in systems biology for revealing conserved biological structures and processes. This study underscores the importance of addressing challenges in data heterogeneity, representation formats, and algorithmic complexity. Key strategies, such as robust preprocessing, seed node selection, and the use of orthology information, are critical for achieving accurate and meaningful alignments. The integration of both local and global alignment methods, combined with advanced computational techniques like graph embeddings and multilayer network analysis, enhances alignment reliability and biological relevance. Moreover, leveraging multiple NA tools and ensuring thorough documentation of workflows promotes reproducibility and transparency in research.

As biological networks grow in complexity and scale, further innovations in NA algorithms and strategies will be essential to manage computational demands and extract nuanced biological insights. By implementing the guidelines and practices discussed in this study, researchers can optimize their NA workflows, facilitating breakthroughs in understanding evolutionary conservation, functional modules, and interspecies relationships. These advancements hold significant promise for applications ranging from disease research to evolutionary biology, contributing to a deeper comprehension of the intricate molecular networks governing life.

## Supporting information

S1 Text This file contains a list of the software tools referenced in this study, along with links to their home pages and summaries of their key features. (PDF)

S2 Text This file explains how systematic documentation promotes clarity and enhances the impact and usability of Network Alignment research. (PDF)
