# Supplementary material for: Ten practical tips and tricks to improve the effectiveness of biological network alignment
Source: PLoS Comput Biol. 2025 Sep 4;21(9):e1013386. doi: 10.1371/journal.pcbi.1013386 (PMC12410751; doi:10.1371/journal.pcbi.1013386)
Supplement: S1 Text — (PDF) [file pcbi.1013386.s001.pdf]

List of Software Tools Mentioned in this Study

Global Alignment Tools

- IsoRank [1] can be obtained freely at `https://cb.csail.mit.edu/mna/`;
- IsoRankN [2] is freely available for download at `https://cb.csail.mit.edu/mna/`;
- MAGNA++ [3] is freely available at `https://www3.nd.edu/~cone/magna++/downloads.html`;
- NETAL [4] is not available for the download at the time of writing;
- HubAlign [5] can be downloaded at `https://github.com/hashemifar/HubAlign`;
- GRAAL [6] available for free download at `http://www0.cs.ucl.ac.uk/staff/natasa/GRAAL/`;
- DANTE [7] is freely available at `https://github.com/pietrocinaglia/dante`;
- DANTEml [8] can be downloaded at `https://github.com/pietrocinaglia/danteml`.

Local Alignment Tools

- AlignMCL [9] is available at `https://github.com/hguzzi/EMB-Align`;
- SPINAL [10] is freely available at `https://code.google.com/archive/p/spinal/downloads`.

Network Alignment Tools integrating deep learning

- DeepAlign [11] can be downloaded at `https://github.com/realbigws/DeepAlign`;
- SUMONA [12] SUMONA's source code and data conversion tools are shared upon request.

Table 1. Summary of Network Alignment Tools. *"?"* represents a feature that has not been possible to evaluate.

**Tool**	**Language**	**Input**	**Output**	**Key Features**
**Global Alignment Tools**				
IsoRank [1]	C++	Graphs (Adjacency Matrices)	Node Correspondences	Uses spectral methods to align networks based on node similarity.
IsoRankN [2]	C++	Graphs	Multiple Network Alignments	Extension of IsoRank, supports multiple network alignment.
MAGNA++ [3]	C++	Graphs (Edge Lists)	Optimized Network Alignment	Uses genetic algorithms to optimize alignment scores.
NETAL [4]	C++	Graphs	Aligned Network	Uses topological similarity for alignment. Not available for download.
HubAlign [5]	C++	Graphs (Adjacency Matrices)	Node Correspondences	Prioritizes hubs (high-degree nodes) in alignment.
GRAAL [6]	C++	Graphs	Aligned Network	Uses graphlet-based similarity measures.
DANTE [7]	Java	Graphs	Optimal Alignment Mapping	Uses integer programming for optimal alignment.
DANTEml [8]	Java	Graphs	Machine Learning-Based Alignment	Machine learning extension of DANTE.
**Local Alignment Tools**				
AlignMCL [9]	Java	Graphs	Clustered Alignments	Uses Markov clustering to detect local alignments.
SPINAL [10]	C++	Graphs	Local Node Correspondences	Uses sequence and topological similarity for local alignment.
**Network Alignment Tools Integrating Deep Learning**				
DeepAlign [11]	C++	Graphs	Deep Learning-Based Alignment	Uses deep learning for similarity learning in network alignment.
SUMONA [12]	?	Graphs	Enhanced Alignment Mapping	Integrates deep learning to improve alignment accuracy.

References

1. Singh R, Xu J, Berger B. Global alignment of multiple protein interaction networks with application to functional orthology detection. Proceedings of the National Academy of Sciences. 2008;105(35):12763–12768.

2. Liao CS, Lu K, Baym M, Singh R, Berger B. IsoRankN: spectral methods for global alignment of multiple protein networks. Bioinformatics. 2009;25(12):i253–i258.

3. Vijayan V, Saraph V, Milenković T. MAGNA++: maximizing accuracy in global network alignment via both node and edge conservation. Bioinformatics. 2015;31(14):2409–2411.

4. Neyshabur B, Khadem A, Hashemifar S, Arab SS. NETAL: a new graph-based method for global alignment of protein–protein interaction networks. Bioinformatics. 2013;29(13):1654–1662.

5. Hashemifar S, Xu J. Hubalign: an accurate and efficient method for global alignment of protein–protein interaction networks. Bioinformatics. 2014;30(17):i438–i444.

6. Kuchaiev O, Przulj N. Global network alignment. Nature Precedings. 2010; p. 1–1.

7. Cinaglia P, Cannataro M. A Method Based on Temporal Embedding for the Pairwise Alignment of Dynamic Networks. Entropy. 2023;25(4). doi:10.3390/e25040665.

8. Cinaglia P, Milano M, Cannataro M. Multilayer network alignment based on topological assessment via embeddings. BMC Bioinformatics. 2023;24(1). doi:10.1186/s12859-023-05508-5.

9. Mina M, Guzzi PH. AlignMCL: Comparative analysis of protein interaction networks through Markov clustering. In: 2012 IEEE International Conference on Bioinformatics and Biomedicine Workshops. IEEE; 2012. p. 174–181.

10. Aladağ AE, Erten C. SPINAL: scalable protein interaction network alignment. Bioinformatics. 2013;29(7):917–924.

11. Nolle T, Seeliger A, Thoma N, Mühlhäuser M. DeepAlign: alignment-based process anomaly correction using recurrent neural networks. In: International conference on advanced information systems engineering. Springer; 2020. p. 319–333.

12. Tuncay EG, Can T. SUMONA: A supervised method for optimizing network alignment. Computational biology and chemistry. 2016;63:41–51.
