# Supplementary material for: Ten practical tips and tricks to improve the effectiveness of biological network alignment
Source: PLoS Comput Biol. 2025 Sep 4;21(9):e1013386. doi: 10.1371/journal.pcbi.1013386 (PMC12410751; doi:10.1371/journal.pcbi.1013386)
Supplement: S2 Text — (PDF) [file pcbi.1013386.s002.pdf]

Network Alignment Example.

Follows a practical example of NA documentation. Suppose that we have to align homo-sapiens and mouse protein-protein interaction (PPI) networks to identify conserved functional modules. The documentation should describe the following relevant aspects.

- Context: The goal is to discover conserved protein complexes between humans and mice, focusing on shared biological processes, such as metabolic pathways.
- Input Data: The human PPI network is sourced from BioGRID, while the mouse network is from STRING. Both networks are cleaned by removing nodes with low connectivity and normalizing them to the node degree.
- Experimental Setup: The researcher uses a heuristic-based Global Network Alignment (GNA) algorithm, such as IsoRank, implemented in Python. The environment includes Python 3.9, NetworkX, and NumPy, executed on a server with 32 CPUs and 128GB RAM.
- Seed Node Selection: Orthologs are identified using OrthoDB. Sequence similarity scores from BLAST are used to prioritize seed pairs, supplemented by functional annotations from Gene Ontology (GO).
- Results: The alignment yields an overall coverage of 78%, with conserved subnetworks including key metabolic enzymes. Visualization in Cytoscape reveals clusters corresponding to known protein complexes.
- Reproducibility: Scripts for preprocessing, alignment, and visualization are shared on GitHub, along with data and configuration files. Limitations, such as incomplete GO annotations, are acknowledged.

Fig 1 shows the main steps of the network alignment documentation workflow.

This example illustrates how systematic documentation ensures clarity and enhances NA research's impact and usability.

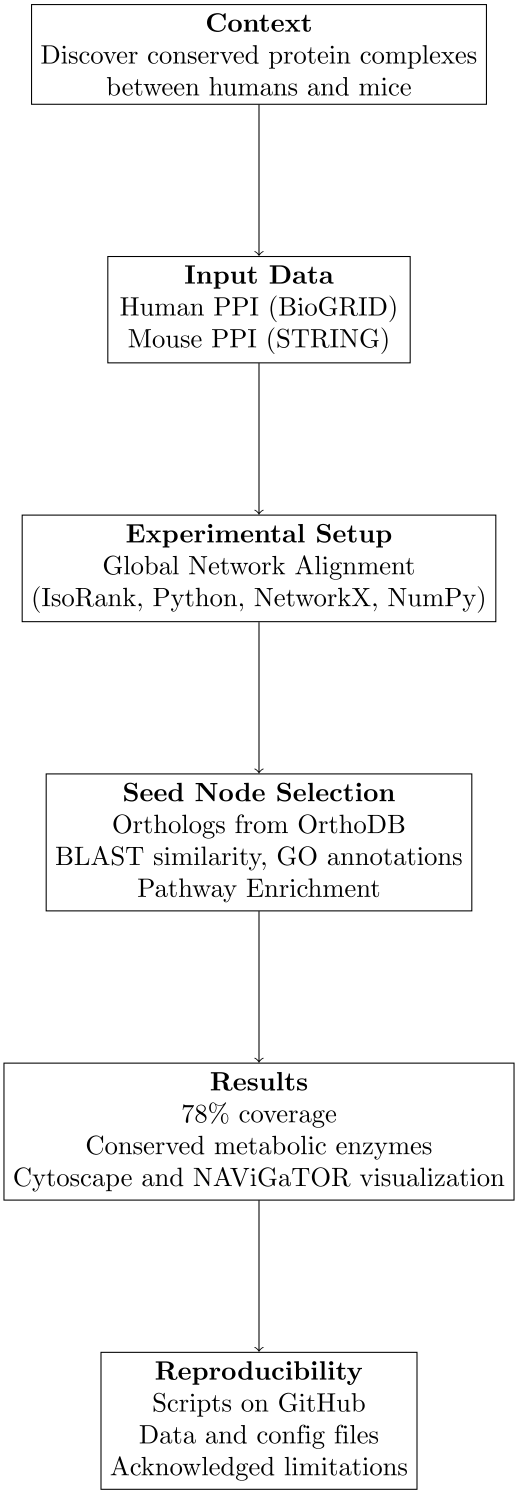

Fig 1. Diagram of Network Alignment Documentation Steps
